# Role of FAAH-Like Anandamide Transporter in Anandamide Inactivation

**DOI:** 10.1371/journal.pone.0079355

**Published:** 2013-11-04

**Authors:** KwanNok Leung, Matthew W. Elmes, Sherrye T. Glaser, Dale G. Deutsch, Martin Kaczocha

**Affiliations:** 1 Department of Biochemistry and Cell Biology, Stony Brook University, Stony Brook, New York, United States of America; 2 Department of Biological Sciences, Kingsborough Community College, Brooklyn, New York, United States of America; 3 Department of Anesthesiology, Stony Brook University, Stony Brook, New York, United States of America; Nathan Kline Institute for Psychiatric Research and New York School of Medicine, United States of America

## Abstract

The endocannabinoid system modulates numerous physiological processes including nociception and reproduction. Anandamide (AEA) is an endocannabinoid that is inactivated by cellular uptake followed by intracellular hydrolysis by fatty acid amide hydrolase (FAAH). Recently, FAAH-like anandamide transporter (FLAT), a truncated and catalytically-inactive variant of FAAH, was proposed to function as an intracellular AEA carrier and mediate its delivery to FAAH for hydrolysis. Pharmacological inhibition of FLAT potentiated AEA signaling and produced antinociceptive effects. Given that endocannabinoids produce analgesia through central and peripheral mechanisms, the goal of the current work was to examine the expression of FLAT in the central and peripheral nervous systems. In contrast to the original report characterizing FLAT, expression of FLAT was not observed in any of the tissues examined. To investigate the role of FLAT as a putative AEA binding protein, FLAT was generated from FAAH using polymerase chain reaction and further analyzed. Despite its low cellular expression, FLAT displayed residual catalytic activity that was sensitive to FAAH inhibitors and abolished following mutation of its catalytic serine. Overexpression of FLAT potentiated AEA cellular uptake and this appeared to be dependent upon its catalytic activity. Immunofluorescence revealed that FLAT localizes primarily to intracellular membranes and does not contact the plasma membrane, suggesting that its capability to potentiate AEA uptake may stem from its enzymatic rather than transport activity. Collectively, our data demonstrate that FLAT does not serve as a global intracellular AEA carrier, although a role in mediating localized AEA inactivation in mammalian tissues cannot be ruled out.

## Introduction

The endocannabinoid anandamide (AEA) is an endogenous ligand for cannabinoid receptors [1]. Through activation of cannabinoid receptors, AEA regulates a plethora of biological processes including nociception, inflammation, reproduction, and gastric motility [2-4]. AEA signaling is terminated through catabolism by the endoplasmic reticulum-localized enzyme fatty acid amide hydrolase (FAAH) [5-7]. Prior to reaching FAAH, lipophilic AEA requires transport through the aqueous cytosol. To date, several intracellular AEA binding proteins have been identified including fatty acid binding proteins (FABPs), Hsp70, and most recently FAAH-like anandamide transporter (FLAT) [8-10]. 

FLAT was reported to be a catalytically-silent truncated (lacking residues 9-76) splice variant of FAAH that is co-expressed with FAAH in mammalian tissues and cell-lines [10], providing a possible mechanism to account for the efficient uptake and inactivation of AEA in diverse tissues. Importantly, inhibition of FLAT potentiated the antinociceptive effects of AEA, suggesting that FLAT may represent a therapeutic target [10]. Because previous reports have shown that the antinociceptive effects of endocannabinoids may be mediated by peripheral cannabinoid receptors [11], we sought to ascertain whether FLAT is expressed in peripheral nerves and whether its antinociceptive effects are mediated through a peripheral endocannabinoid mechanism. 

## Materials and Methods

### Chemicals

AEA, URB597, FP-3845, and MAFP were from Cayman Chemical (Ann Arbor, MI), fatty acid-free bovine serum albumin was from Sigma, [^14^C]AEA (arachidonoyl-[1-^14^C]ethanolamide, 60 mCi/mmol) was provided by the Drug Supply Program at the National Institute on Drug Abuse. 

### Cell culture and transfections

HeLa and HEK-293 cells were grown in DMEM supplemented with 10% fetal bovine serum, 100 units/ml penicillin/streptomycin, 2 mM L-glutamine, and 1 mM sodium pyruvate. The cells were transfected with FAAH, FLAT, green fluorescent protein (GFP)-tagged FAAH (FAAH-GFP), or plasma membrane-localized GFP (PM-GFP) using the GenJet Plus reagent (SignaGen, Rockville, MD) according to the manufacturer’s instructions. PM-GFP was previously described [12] and was kindly provided by Deborah Brown (Stony Brook University). 

### Immunofluorescence

HeLa cells were transfected with FAAH-FLAG, FLAT-FLAG, PM-GFP, or FAAH-GFP. Immunolocalization of proteins was performed exactly as described [9]. Following fixation and permeabilization, cells were incubated with rabbit anti-FABP5 (1:500) (BioVendor R&D, Candler, NC), mouse anti-FAAH (1:200) (Abcam, item #ab54615, immunogen: amino acids 480-580 of human FAAH), followed by donkey anti-rabbit 594 or goat anti-mouse 594 (Molecular Probes) antibodies. The images were acquired using a Zeiss LSM 510 META NLO Two-Photon Laser Scanning Microscope. 

### Western blotting

Western blotting was carried out exactly as described [9]. Membranes were probed with FAAH (1:400), β-actin (1:20,000) (Abcam), or calnexin (1:5000) (Novus Biologicals) and developed using the Immun-star HRP substrate (Bio-Rad) and exposed to film.

### Reverse transcription-polymerase chain reaction (RT-PCR)

RT-PCR was carried out as described [13]. Briefly, cDNA was prepared from RNA using the Superscript III first strand synthesis system (Invitrogen) and subjected to PCR using LongAmp Taq DNA polymerase (New England Biolabs) and primers specific for the open reading frame of FAAH. The following primers were used: forward 5’-ATGGTGCTGAGCGAAGTGTG-3’ and reverse 5’-AAGATGGCCGCTTTTCAGG-3’. The cycling conditions were as follows: denaturation at 94°C for 30 sec, annealing at 58°C for 30 sec, and extension at 65°C for 2 min for a total of 35 cycles.

### Enzyme assays

FAAH and FLAT activity was determined as described previously [9]. Briefly, cell homogenates were pretreated for 10 min with vehicle (1% ethanol) or inhibitors and subsequently incubated with 30 μM [^14^C]AEA at 37°C for 5-30 min, ensuring that substrate conversion remained at ~10-15%. Reactions were stopped with two volumes of chloroform:methanol (1:1) and the methanol phase was counted using a Beckman LS6500 scintillation counter. 

### AEA uptake

Inhibition of AEA uptake into cells was performed exactly as described [9]. FAAH, FLAT, or vehicle transfected HeLa cells were incubated with 100 nM [^14^C]AEA for 5 min or 3 sec at 37°C. AEA uptake was quantified as previously described [9].

### Statistics

Results represent means ± SE of at least three independent experiments performed in triplicate. Statistical significance was determined using two-tailed unpaired t tests or one-way ANOVA followed by Dunnett’s post hoc analysis against the corresponding controls.

## Results

### Expression of FLAT in mouse tissues

**Figure 1:**
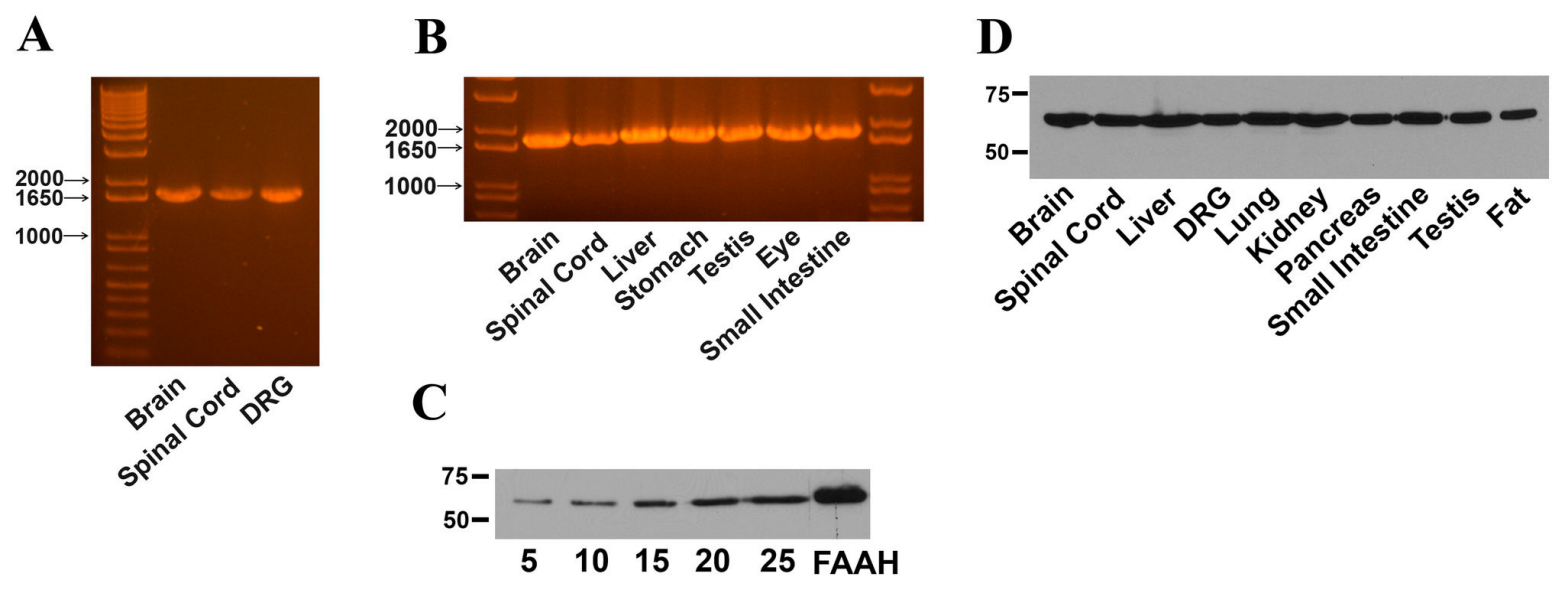
Expression of FAAH and FLAT in mouse tissues. (A) RT-PCR analysis of FAAH and FLAT expression in mouse brain, spinal cord, and L3-L5 DRG. Transcripts representing full-length FAAH (~1740 base pair fragments) were found in all tissues examined. In contrast, FLAT (~1540 base pair fragment) was not detected in any of the tissues. (B) RT-PCR analysis of FAAH and FLAT expression in mouse tissues. (C) Western blot demonstrating the expression of FAAH (~63 kDa) in mouse brain. Increasing the amount of brain tissue from 5 to 25 µg did not reveal the presence of FLAT, which has an expected molecular weight of ~56 kDa. FAAH-transfected HeLa cells are also shown. (D) Western blot of FAAH expression in a panel of mouse tissues.

Antinociceptive effects of endocannabinoids are mediated by both centrally and peripherally expressed cannabinoid receptors [11,14-16]. While FAAH inhibitors reduce nociception through central and peripheral FAAH inhibition [14,17], it is currently not known whether FLAT regulates nociception at peripheral or central sites. To address this, we examined the expression of FLAT in mouse brain, spinal cord, and L3-L5 dorsal root ganglia (DRG). Surprisingly, RT-PCR analysis failed to detect FLAT in these tissues while FAAH expression was readily observed (Figure 1A). We extended our analysis to other peripheral tissues but again failed to detect FLAT (Figure 1B). To determine whether FLAT expression could be observed at the protein level, we performed western blotting using an antibody raised against the C-terminus of FAAH (see Methods), which is expected to also detect FLAT. Similar to the RT-PCR results, our analysis revealed the presence of FAAH but failed to detect FLAT in mouse brain regardless of the protein concentration used (Figure 1C). Similar results were observed in other mouse tissues (Figure 1D).

**Figure 2:**
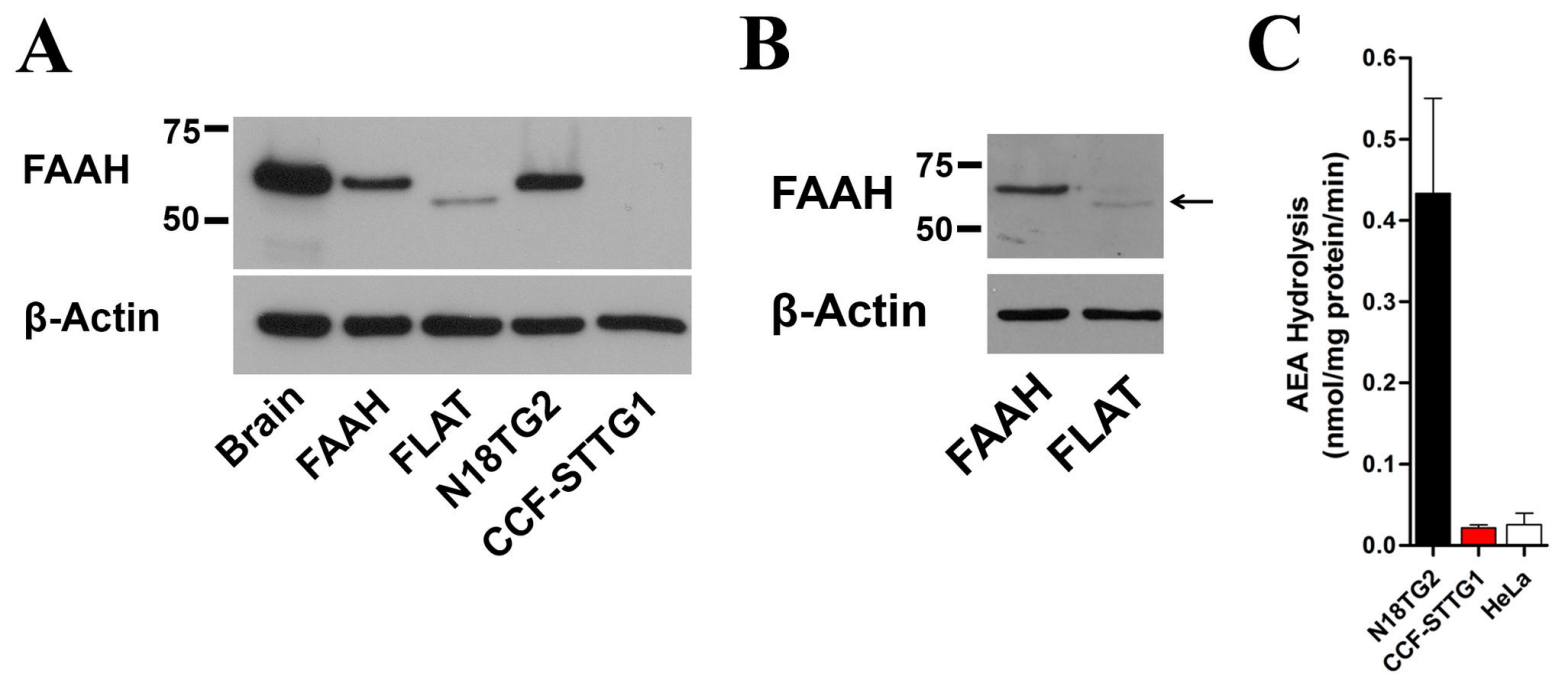
Expression of FAAH and FLAT in cell-lines. (A) Western blot of FAAH and FLAT expression in brain, HeLa cells transfected with FAAH (0.15 µg) or FLAT (1.5 µg), and in N18TG2 neuroblastoma and CCF-STTG1 astrocytoma cells. (B) Expression of FAAH and FLAT following transfection into HEK-293 cells. (C) AEA hydrolysis in homogenates of N18TG2, CCF-STTG1, and HeLa cells (n = 3).

To permit further analyses of FLAT, we constructed rat FLAT by removing residues 9-76 from FAAH. In contrast to FAAH, FLAT was relatively poorly expressed upon transfection into HeLa or HEK-293 cells (Figure 2A and B). Attempts to increase FLAT expression using alternate cell-lines did not improve expression levels (data not shown), suggesting that FLAT may be an intrinsically unstable protein. Using transfected FLAT as a guide, we confirmed that the mouse brain lacks FLAT expression (Figure 2A). Analysis of FLAT expression was extended to two cell-lines, mouse N18TG2 neuroblastoma and human CCF-STTG1 astrocytoma cells. In agreement with previous work [7], FAAH expression and activity was observed in N18TG2 cells but was absent in CCF-STTG1 cells (Figure 2A and C). In contrast, FLAT expression was not observed in either cell-type.

### Functional Analysis of FLAT

**Figure 3:**
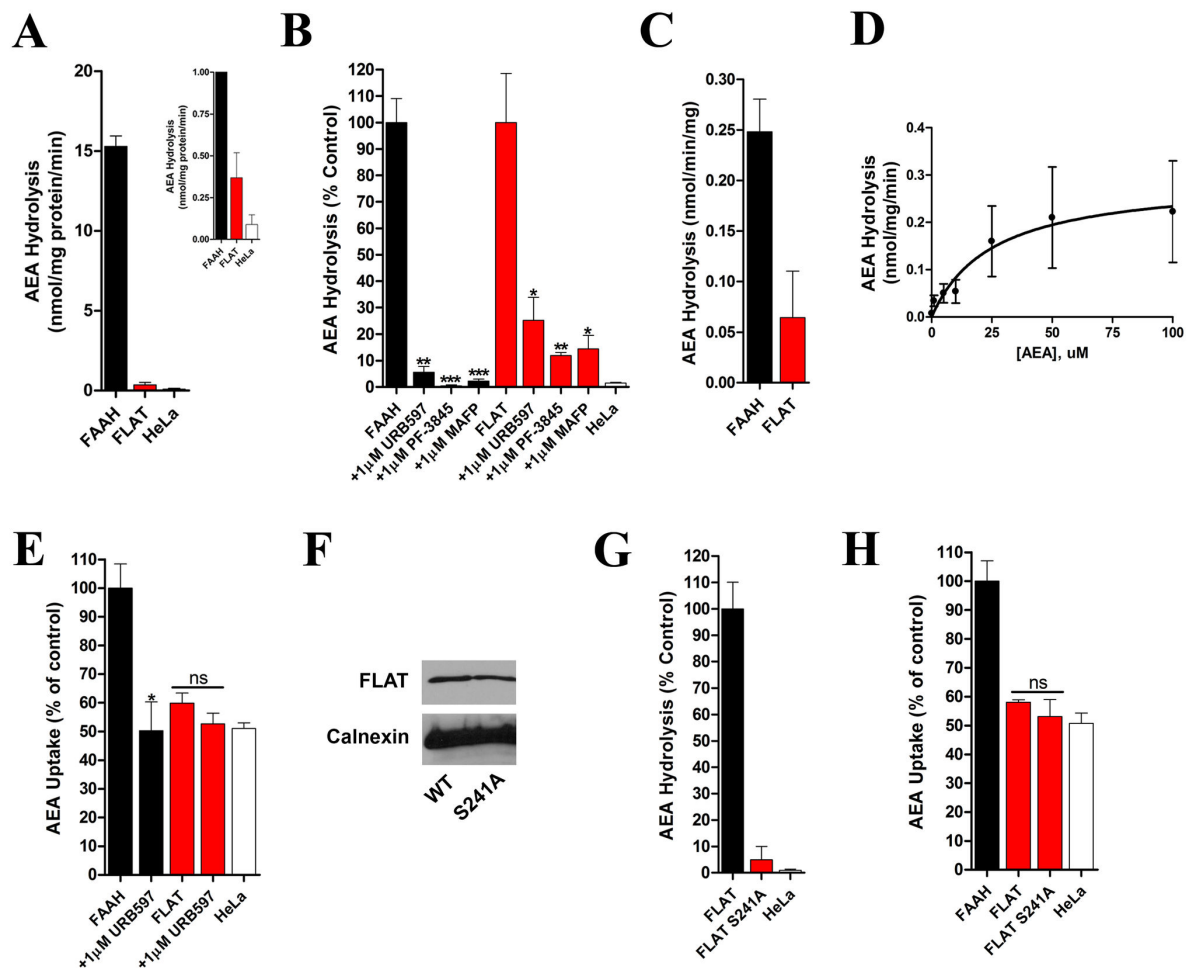
Effect of FLAT upon AEA hydrolysis and uptake. (A) AEA hydrolysis by HeLa cell homogenates transfected with FAAH or FLAT. Inset: Close up of AEA hydrolysis by FLAT-transfected and vector-transfected HeLa homogenates (n = 3). (B) Inhibition of FAAH and FLAT by URB597, PF-3845, and MAFP. *, p < 0.05; **, p < 0.01; ***, p < 0.001 (n = 3-5). (C) AEA (100 nM) hydrolysis by FLAT normalized to its expression level relative to FAAH (n = 3). (D) FLAT hydrolyzes AEA (0.1-100 µM) with a K_m_ of 25.3 ± 14.2 µM and V_max_ of 0.29 ± 0.13 nmol/mg/min (n = 3-5). (E) AEA uptake in HeLa cells transfected with FAAH or FLAT in the absence or presence of URB597. *, p < 0.05 (n = 3). (F) Western blot showing equal expression of WT and S241A FLAT in HeLa cells. (G) Enzymatic activity of WT and S241A FLAT. (H) AEA uptake in HeLa cells expressing WT or S241A FLAT (n = 3).

**Figure 4:**
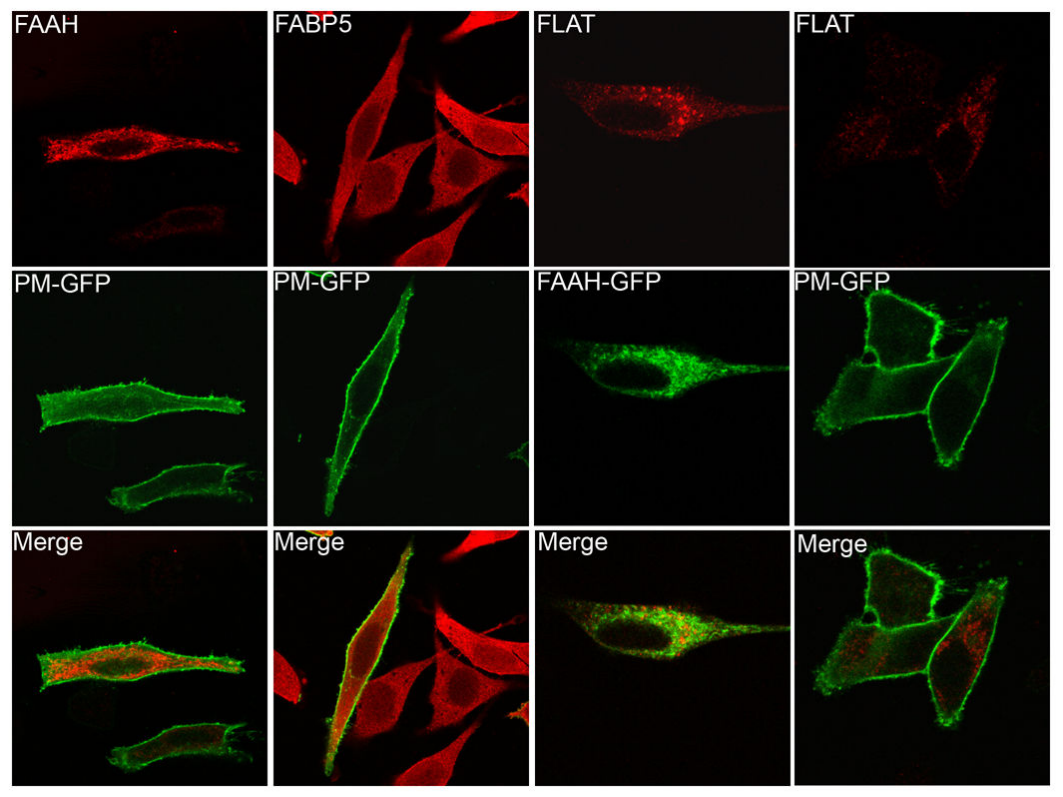
Localization of FLAT in HeLa cells. Subcellular distribution of FAAH, FLAT, and FABP5 in cells. FAAH and FLAT do not co-localize with the plasma membrane marker PM-GFP while FABP5 does. In contrast, FAAH and FLAT are distributed on intracellular membranes.

The catalytic activity of FLAT was also examined. Despite its low expression, FLAT possessed detectable enzymatic activity that was sensitive to inhibition by the FAAH inhibitors URB597, PF-3845, and MAFP, although complete inhibition was never observed (Figure 3A and B). Normalization of FLAT activity to its expression level relative to FAAH revealed that FLAT displays ~20% of the catalytic activity of FAAH at physiological substrate concentrations (Figure 3C). Further kinetic analysis demonstrated that FLAT hydrolyzes AEA with a K_m_ of 25.3 ± 14.2 µM and V_max_ of 0.29 ± 0.13 nmol/mg/min (Figure 4D). We next examined whether FLAT modulates AEA cellular uptake given that AEA uptake is known to be potentiated in cells expressing catalytically competent FAAH, whose enzymatic activity maintains an inward AEA concentration gradient [13,18-20]. Overexpression of FLAT modestly potentiated AEA uptake and although the inclusion of URB597 did not reduce AEA uptake, a trend for reduced accumulation was observed (Figure 3E). Indeed, the magnitude of FLAT-potentiated AEA uptake was considerably lower than that observed with FAAH, presumably a result of its low expression level. To confirm that FLAT potentiates AEA uptake by hydrolyzing internalized AEA, we mutated its catalytic serine to alanine. The expression levels of FLAT and its S241A mutant (using labeling nomenclature from FAAH) were similar (Figure 3F). As expected, FLAT bearing the S241A mutation was catalytically inactive (Figure 3G). Overexpression of wild-type FLAT in cells potentiated AEA transport and this effect was lower in cells expressing S241A FLAT, although again it did not reach statistical significance (Figure 3H). 

Lastly, we explored the subcellular localization of FLAT. FAAH localizes to the endoplasmic reticulum and does not co-localize with the plasma membrane marker PM-GFP (Figure 4) [7]. Similar to FAAH, FLAT was distributed on intracellular membranes and did not co-localize with PM-GFP (Figure 4). In contrast, the intracellular AEA binding protein FABP5 was distributed throughout the cytoplasm and regions of co-localization with PM-GFP were observed. 

## Discussion

Signaling lipids often depend on specialized transport systems to ensure efficient ligand delivery and disposal. For example, prostaglandins are thought to utilize plasma membrane-localized transporters to traverse biological membranes [21,22]. In contrast to such charged lipids, endocannabinoids are uncharged and more lipophilic and readily cross synthetic [23,24] and biological membranes [20,25,26], although the existence of an endocannabinoid transmembrane transporter has also been proposed [27]. In contrast, the aqueous cytosol presents a diffusional barrier that can be overcome through carrier-assisted intracellular transport. AEA uptake is observed in a wide array of cell types suggesting that proteins that mediate AEA uptake should exhibit broad tissue distribution (for review, see 28,29). FABPs and Hsp70s fit such a criterion as they are found in numerous tissues and cell-types [30,31]. FLAT was also reported as a widely expressed intracellular binding protein that selectively transports AEA and likewise fits such criteria [10]. 

Endocannabinoids produce analgesia through central and peripheral mechanisms [2,11,14]. Because pharmacological inhibition of FLAT potentiated AEA-mediated analgesia *in vivo* [10], the primary motivation for this manuscript was to investigate the expression of FLAT in peripheral nerves. Using RT-PCR and western blotting, we were unable to detect FLAT expression in DRGs nor in other tissues examined, possibly suggesting that FLAT may exhibit a low tissue expression profile that is below the detection limit of these approaches. These data are in contrast to the findings of Fu et al. who observed robust FLAT expression throughout the brain [10]. The reason for this discrepancy is unclear. One possibility is the use of different antibodies (i.e., monoclonal antibodies in this manuscript vs. polyclonal FAAH antibodies by Fu et al.). Confidence in the specificity of our approach is strengthened by the finding that FAAH was not detected in a cell-line known to be devoid of FAAH expression and activity. Furthermore, the presence of a single band corresponding to FAAH in western blots is supported by previous studies [6,32-36]. 

FLAT was reported to be a catalytically silent splice variant of FAAH [10]. Our present data are mostly in agreement with these results given the extremely low catalytic activity of FLAT. Although our results suggest that this residual activity may contribute towards AEA uptake, our analysis was hampered by the low expression level of FLAT and did not yield a definitive answer. Pharmacological inhibition of FLAT with ARN272 was shown to potentiate AEA signaling and produces analgesia [10]. Therefore, how can one reconcile our inability to detect FLAT with such profound antinociceptive effects observed following FLAT inhibition? One possibility is that FLAT may be strategically expressed in brain regions that modulate pain such as the periaqueductal gray, a region known to express FAAH [37,38]. Such a low level and localized expression pattern may have eluded our detection, which focused upon global FLAT expression. Alternatively, ARN272 may target proteins distinct from FLAT that regulate AEA inactivation and AEA-mediated analgesia. 

There are numerous outstanding questions regarding this intriguing protein that merit further exploration. For example, FAAH is a promiscuous enzyme that hydrolyzes numerous N-acylethanolamines in addition to AEA [39-41]. In contrast, FLAT has been reported to display an unprecedented selectivity for AEA [10]. Similarly, while FAAH does not interact with putative AEA transport inhibitors bearing oleoyl acyl chains such as OMDM1 ((S)-N-oleoyl tyrosinol) [20,42], FLAT does. Collectively, our results demonstrate that FLAT is not widely expressed in mammalian tissues, indicating that it is unlikely to serve as a global intracellular endocannabinoid carrier but may instead mediate regional AEA inactivation.
